# Association between osteoarthritis with Parkinson’s disease in the US (NHANES 2011–2020)

**DOI:** 10.3389/fnins.2024.1393740

**Published:** 2024-08-21

**Authors:** Yang Liu, Xue Zhou, Chunhai Chen, Xuefeng Li, Ting Pan, Ziqi Liu, Dalong Wu, Xinhua Chen

**Affiliations:** ^1^Changchun University of Chinese Medicine, Changchun, China; ^2^The Affiliated Hospital to Changchun University of Chinese Medicine, Changchun, China

**Keywords:** Parkinson’s disease, osteoarthritis, cross-sectional study, NHANES, neurodegenerative disease

## Abstract

**Objected:**

To evaluate the association between osteoarthritis (OA) and Parkinson’s disease (PD) in adults in the United States.

**Methods:**

Using 2011–2020 NHANES data, a cross-sectional study of 11,117 adults over the age of 40 was conducted. Univariate logistic regression and multivariate logistic regression were used to analyze the relationship between arthritis and PD. In addition, stratified analysis was used to examine whether the relationship between arthritis and PD was interactive with age, gender, race, education, BMI.

**Results:**

In this study, a total of 11,117 participants were included, and we found that osteoarthritis was positively correlated with the development of PD compared with non-arthritis patients [1.95 (1.44 ~ 2.62)] (*p* < 0.001). After adjusting the covariates, the results are still stable.

**Conclusion:**

PD patients were positively correlated with OA. Among people with OA, there was a 95% increased risk of PD compared to people without arthritis. Therefore, when treating OA, attention should be paid to the increased risk of PD. In the meantime, further studies are needed to explore the link between OA and PD patients.

## Introduction

Parkinson’s disease (PD) is a prevalent degenerative disorder of the central nervous system, ranking second only to Alzheimer’s disease (Hirtz et al., 2007). Epidemiological studies indicate that the incidence of PD rises with age (Savica et al., 2016). The primary clinical manifestations include motor symptoms such as resting tremors, muscle stiffness, bradykinesia, and postural instability (Bledsoe et al., 2023). Characteristic pathological features encompass dopaminergic neuron degeneration and loss, *α*-synuclein (*α*-syn) aggregation, and the presence of Lewy bodies (Jankovic and Tan, 2020). Although the pathogenesis of PD remains elusive, emerging evidence suggests a potential role of inflammation in the disease. Various studies have demonstrated that heightened levels of inflammatory mediators can activate microglia, contributing to dopaminergic neuron degeneration (Lücking et al., 2000; Perry, 2004). Neuroinflammation observed in PD patients may also influence the onset or progression of chronic neurodegenerative conditions (Lücking et al., 2000; Perry, 2004).

Arthritis is an inflammatory disease that affects the joints and surrounding tissues of the body. Studies have shown a rise in its incidence and prevalence over the years (The Lancet, 2017). The types of arthritis include osteoarthritis (OA), rheumatoid arthritis (RA), psoriatic arthritis (PsA), and other forms (Tang, 2019). These types are categorized as inflammatory joint diseases with varying causes (Frangos and Maret, 2020). In the case of OA, inflammation, whether local or systemic, can lead to joint and bone damage, with specific inflammatory factors such as IL-1*α*, tumor necrosis factor (TNF-*α*), and C-reactive protein playing a significant role in its development (Hn and Vb, 2015). Similarly, in patients with Parkinson’s disease (PD), there are also elevated levels of related inflammatory factors, which differ significantly from those seen in PD alone (Liu T.-W. et al., 2022).

In patients, *α*-syn can activate inflammatory bodies peripherally, thus triggering peripheral inflammation (Tan et al., 2020). Peripheral inflammation is also considered an essential factor in PD pathogenesis (Fan et al., 2020). The widespread peripheral inflammation may indicate an interaction between osteoarthritis and PD. Studies have shown that C-reactive protein and erythrocyte sedimentation rate are correlated with the prevalence of PD, and they are also biomarkers of arthritis (Fardell et al., 2021; Qu et al., 2023). Additionally, non-steroidal anti-inflammatory drugs (NSAIDs) are commonly used in osteoarthritis treatment, and prolonged use of these medications can lower the risk of PD (Schiess, 2003). Studies have demonstrated that NSAIDs can help maintain neuronal integrity (Casper et al., 2000; Teema et al., 2016). Therefore, it is plausible to assume an association between OA patients taking NSAIDs and PD. However, the results of studies investigating the link between osteoarthritis and Parkinson’s disease remain inconclusive.

The existing research on the correlation between osteoarthritis and Parkinson’s disease is inconclusive. To further examine this relationship, we conducted a study on a significant sample size sourced from the US National Health and Nutrition Examination Survey (NHANES). This study is believed to be the first to utilize NHANES data in investigating the potential link between osteoarthritis and PD risk.

## Materials and methods

### Data source

**Figure 1:**
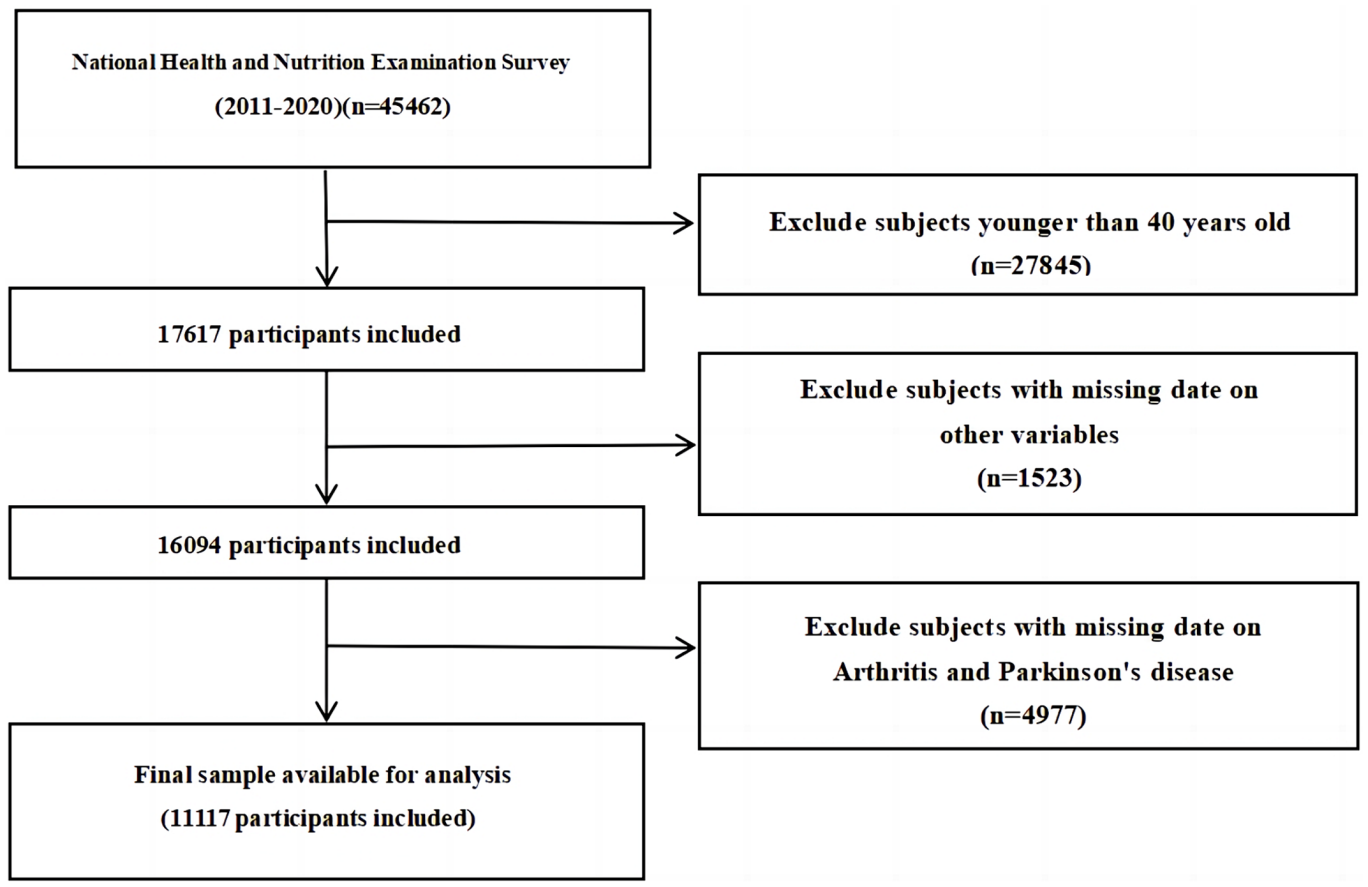
Flow diagram of the sample selection from the National Health and Nutrition Examination Survey (NHANES) 2011–2020.

The study utilized data from the NHANES database spanning from 2011 to 2020. The NHANES Database, available at https://www.cdc.gov/nchs/nhanes/index.htm, is a research program aimed at evaluating the health and nutrition status of individuals in the United States, covering various populations and health topics. A decade’s worth of data was collected from this database to construct the dataset for the study. Initially, individuals aged 40 and above were considered for exclusion, followed by the exclusion of participants with incomplete personal information. The specific exclusion criteria employed in this study are detailed in Figure 1. Ultimately, the study included a total of 11,117 NHANES participants.

### Assessment of Parkinson’s disease

In the NHANES database, in conjunction with previous literature (Liu S.-Y. et al., 2022; Liu et al., 2023), individuals taking specific Parkinson’s drugs were classified as having Parkinson’s disease. This classification was determined based on participants’ responses regarding prescription medications. It is important to note that this method is constrained by the medications and codes available in NHANES, requiring individuals to be actively receiving Parkinson’s disease medication to be categorized as having the disease. Conversely, those who were not taking the specified medications were classified as not having Parkinson’s disease.

### Assessment of arthritis

Arthritis diagnosis in the survey was determined based on two key questions. The first question asked participants if a doctor had ever diagnosed them with arthritis. Those who responded “No” were categorized as non-arthritis cases. For those who answered “Yes,” a follow-up question inquired about the specific type of arthritis diagnosed. Participants were further classified into osteoarthritis, rheumatoid arthritis, psoriatic arthritis, or other types based on their responses to the second question.

### Measurements of other covariates

According to previous research and clinical experience, the key variables examined in this research included demographic factors, personal medical history, and chronic conditions (Zeng et al., 2023, 2024). Demographic factors encompassed age, sex (female, male), race (Mexican American, other Hispanic, non-Hispanic white, non-Hispanic black, other race), marital (married, living with a partner, separated, widowed, divorced, never married), and education (≤9, 9–12, ≥12). Prior studies have suggested that nicotine may serve as a protective factor for Parkinson’s disease by enhancing the survival of dopaminergic neurons (Liu et al., 2023), hence smoking was considered a significant covariate. Participants were queried about their history of smoking at least 100 cigarettes in their lifetime, with a ‘Yes’ response indicating a smoker and ‘No’ indicating a non-smoker. Chronic diseases of interest included diabetes, hypertension, hyperlipidemia, heart disease, stroke, and cancer. Body Mass Index (BMI) was utilized as a standard measure of obesity, with weight and height being measured by trained health professionals. BMI was calculated as weight (kg) divided by height squared (m^2^), and both age and BMI were treated as continuous variables.

### Statistical analyses

SPSS was utilized for data extraction and cleaning, while the Nhanes R package in R software (v4.2.1) and FreeStatistics (v1.9) were employed for data analysis (Zeng et al., 2024). Categorical variables were presented as frequencies and percentages, whereas continuous variables were depicted as means and standard deviations. To investigate the relationship between arthritis and PD, a multivariate logistic regression model and univariate logistic analysis were conducted, adjusting for potential confounders. Several models were utilized: Model I with no covariate adjustments, Model II with adjustments for sex, age, race, marital, education, and BMI, Model III with the addition of smoking to the Model II covariates, and Model IV which further adjusted for diabetes, hypertension, hyperlipidemia, heart disease, stroke, and cancer. Univariate regression analysis revealed associations between all these factors and PD. Subgroup analyses were then performed based on age, sex, race, education, and BMI.

## Results

### Characteristics of participants

The final analysis involved 11,117 participants from NHANES cycles 2011–2020, with 5,150 reporting arthritis, including 2,318 patients with osteoarthritis. Table 1 details the participants’ demographic characteristics, smoking history, and chronic complications, revealing differences in the prevalence of conditions like heart disease, stroke, cancer, diabetes, hypertension, and hyperlipemia among the groups. The prevalence of non-arthritis, osteoarthritis, rheumatoid arthritis, and other arthritis among PD patients were 1.8, 4.0, 2.7, and 3.6% respectively, suggesting a correlation between osteoarthritis and PD.

**Table 1:** Data characteristics of the participants.

Variables	Total (*n* = 11,117)	Non-arthritis (*n* = 5,967)	OA (*n* = 2,318)	RA (*n* = 979)	Other (*n* = 1853)
Age, *n* (%)					
<64	6,252 (56.2)	3,820 (64)	1,021 (44)	508 (51.9)	903 (48.7)
>64	4,865 (43.8)	2,147 (36)	1,297 (56)	471 (48.1)	950 (51.3)
Sex, *n* (%)					
Male	5,118 (46.0)	3,087 (51.7)	819 (35.3)	414 (42.3)	798 (43.1)
Female	5,999 (54.0)	2,880 (48.3)	1,499 (64.7)	565 (57.7)	1,055 (56.9)
Race, *n* (%)					
Mexican American	1,087 (9.8)	641 (10.7)	171 (7.4)	113 (11.5)	162 (8.7)
Other Hispanic	1,121 (10.1)	628 (10.5)	170 (7.3)	101 (10.3)	222 (12)
Non-Hispanic White	4,725 (42.5)	2,287 (38.3)	1,352 (58.3)	326 (33.3)	760 (41)
Non-Hispanic Black	2,771 (24.9)	1,453 (24.4)	415 (17.9)	347 (35.4)	556 (30)
Other Race	1,413 (12.7)	958 (16.1)	210 (9.1)	92 (9.4)	153 (8.3)
Marital, *n* (%)					
Married	6,611 (59.5)	3,794 (63.6)	1,342 (57.9)	515 (52.6)	960 (51.8)
Unmarried	4,506 (40.5)	2,173 (36.4)	976 (42.1)	464 (47.4)	893 (48.2)
Education, *n* (%)					
≤9	1,170 (10.5)	614 (10.3)	164 (7.1)	135 (13.8)	257 (13.9)
9–12	3,954 (35.6)	2005 (33.6)	780 (33.6)	414 (42.3)	755 (40.7)
≥12	5,993 (53.9)	3,348 (56.1)	1,374 (59.3)	430 (43.9)	841 (45.4)
BMI, *n* (%)					
Under normal weight	2,485 (22.4)	1,541 (25.8)	447 (19.3)	185 (18.9)	312 (16.8)
Overweight	3,626 (32.6)	2068 (34.7)	684 (29.5)	303 (30.9)	571 (30.8)
Obese	5,006 (45.0)	2,358 (39.5)	1,187 (51.2)	491 (50.2)	970 (52.3)
Smoking, *n* (%)					
No	5,314 (47.8)	2,629 (44.1)	1,201 (51.8)	506 (51.7)	978 (52.8)
Yes	5,803 (52.2)	3,338 (55.9)	1,117 (48.2)	473 (48.3)	875 (47.2)
Heart disease, *n* (%)					
No	891 (8.0)	374 (6.3)	247 (10.7)	93 (9.5)	177 (9.6)
Yes	10,226 (92.0)	5,593 (93.7)	2071 (89.3)	886 (90.5)	1,676 (90.4)
Stroke, *n* (%)					
No	821 (7.4)	348 (5.8)	203 (8.8)	106 (10.8)	164 (8.9)
Yes	10,296 (92.6)	5,619 (94.2)	2,115 (91.2)	873 (89.2)	1,689 (91.1)
Cancer, *n* (%)					
No	1904 (17.1)	822 (13.8)	532 (23)	183 (18.7)	367 (19.8)
Yes	9,213 (82.9)	5,145 (86.2)	1786 (77)	796 (81.3)	1,486 (80.2)
Diabetes, *n* (%)					
No	2,891 (26.0)	1,464 (24.5)	588 (25.4)	304 (31.1)	535 (28.9)
Yes	7,787 (70.0)	4,286 (71.8)	1,629 (70.3)	632 (64.6)	1,240 (66.9)
Border	439 (3.9)	217 (3.6)	101 (4.4)	43 (4.4)	78 (4.2)
Hypertension, *n* (%)					
No	6,999 (63.0)	3,427 (57.4)	1,580 (68.2)	694 (70.9)	1,298 (70)
Yes	4,118 (37.0)	2,540 (42.6)	738 (31.8)	285 (29.1)	555 (30)
Hyperlipemia, *n* (%)					
No	6,169 (55.5)	3,053 (51.2)	1,435 (61.9)	556 (56.8)	1,125 (60.7)
Yes	4,948 (44.5)	2,914 (48.8)	883 (38.1)	423 (43.2)	728 (39.3)
PD, *n* (%)					
No	10,827 (97.4)	5,861 (98.2)	2,226 (96)	953 (97.3)	1787 (96.4)
Yes	290 (2.6)	106 (1.8)	92 (4)	26 (2.7)	66 (3.6)

OA, osteoarthritis; RA, rheumatic arthritis; BMI, body mass index; PD, Parkinson’s disease.

### Association between arthritis and PD

In the overall study population of PD patients, univariate regression analysis indicated that PD was positively associated with race, marital, smoking, stroke, osteoarthritis and other arthritis-related factors (*p* < 0.05) (Table 2). Specifically, Non-Hispanic White exhibited a relatively lower risk of PD compared to Mexican American. Furthermore, the prevalence of PD was lower among married individuals compared to their unmarried counterparts (OR: 1.32, 95% CI: 1.05 to 1.67). Patients with a history of stroke demonstrated a significantly higher risk of developing PD. Compared to patients without arthritis, those with OA and other forms of arthritis exhibited a higher likelihood of developing PD, with OR and 95% CI of 2.29 (1.72–3.03) and 2.04 (1.5–2.79), respectively. Our study identified a statistically significant positive correlation between the presence of OA and other types of arthritis and the incidence of PD (Table 2).

**Table 2:** Univariate arthritis analysis.

Variable	OR_95CI	*p*_value
Age:<64 vs. >64	1.21 (0.95 ~ 1.52)	0.117
Sex: Female vs. Male	1.2 (0.95 ~ 1.52)	0.136
Race: ref. = Mexican American		
Other Hispanic	1.25 (0.7 ~ 2.23)	0.443
Non-Hispanic White	2.05 (1.3 ~ 3.23)	0.002*
Non-Hispanic Black	0.74 (0.44 ~ 1.27)	0.276
Other Race	0.69 (0.37 ~ 1.29)	0.249
Marital: Married vs. Unmarried	1.32 (1.05 ~ 1.67)	0.019*
Education: ref. ≤ 9		
9–12	1.09 (0.72 ~ 1.66)	0.671
≥12	1.04 (0.69 ~ 1.55)	0.857
BMI	0.99 (0.98 ~ 1.01)	0.403
Smoking: No vs. Yes	0.75 (0.59 ~ 0.95)	0.016*
Heart Disease: No vs. Yes	0.78 (0.53 ~ 1.15)	0.208
Stroke: No vs. Yes	0.52 (0.36 ~ 0.73)	<0.001**
Cancer: No vs. Yes	0.94 (0.7 ~ 1.28)	0.713
Diabetes: ref. = No		
Yes	1.03 (0.79 ~ 1.35)	0.826
Border	0.61 (0.28 ~ 1.33)	0.212
Hypertension: No vs. Yes	0.99 (0.78 ~ 1.27)	0.958
Hyperlipemia: No vs. Yes	1.1 (0.87 ~ 1.39)	0.407
Arthritis: ref. = Non-arthritis		
OA	2.29 (1.72 ~ 3.03)	<0.001**
RA	1.51 (0.98 ~ 2.33)	0.064
Other	2.04 (1.5 ~ 2.79)	<0.001**

OR, odds ratio; CI, confdence interval.

*p < 0.05, **p < 0.001. *p* < 0.05 was considered statistically significant.

### Multivariable logistics regression analysis

Table 3 displays the logistic regression findings concerning the relationship between arthritis and PD. The unadjusted model (Model I) indicated an elevated risk of osteoarthritis and other forms of arthritis in relation to PD [2.29 (1.72–3.03), 2.04 (1.5–2.79)]. Upon adjusting for age, sex, race, marital, education, and BMI (Model II), the association between PD and osteoarthritis as well as other arthritis remained significant [1.94 (1.44–2.61), 1.96 (1.42–2.69)]. Further inclusion of smoking in the model (Model III) did not alter the significant relationship between PD and osteoarthritis or other arthritis [1.91 (1.42–2.57), 1.93 (1.4–2.66)]. Finally, after accounting for additional chronic conditions like diabetes, hypertension, hyperlipidemia, heart disease, stroke, and cancer (Model IV), the notable association between PD and osteoarthritis and other arthritis persisted [1.95 (1.44–2.62), 1.96 (1.42–2.71)]. These results suggest a robust and stable correlation between the occurrence of osteoarthritis and Parkinson’s disease, with a significant statistical significance (*p* < 0.001).

**Table 3:** Multivariate logistic regression analysis of the correlation between arthritis and PD.

	Model I	Model II	Model III	Model IV
	OR (95% CI)	OR (95% CI)	OR (95% CI)	OR (95% CI)
Non-arthritis	1 (Ref)	1 (Ref)	1 (Ref)	1 (Ref)
OA	2.29 (1.72 ~ 3.03)**	1.94 (1.44 ~ 2.61)**	1.91 (1.42 ~ 2.57)**	1.95 (1.44 ~ 2.62)**
RA	1.51 (0.98 ~ 2.33)	1.54 (0.99 ~ 2.39)	1.52 (0.98 ~ 2.36)	1.5 (0.96 ~ 2.34)
Other	2.04 (1.5 ~ 2.79)**	1.96 (1.42 ~ 2.69)**	1.93 (1.4 ~ 2.66)**	1.96 (1.42 ~ 2.71)**
P for trend	<0.001	<0.001	<0.001	<0.001

Model I: no adjusted; Model II: adjusted for age + sex + race + marital + education + BMI; Model III: Model II+ smoking; Model IV: Model III + diabetes + hypertension + hyperlipemia + heart disease + stroke + cancer. *p < 0.05, **p < 0.001. *p* < 0.05 was considered statistically significant.

### Subgroup analyses of factors influencing the association between arthritis and PD

**Figure 2:**
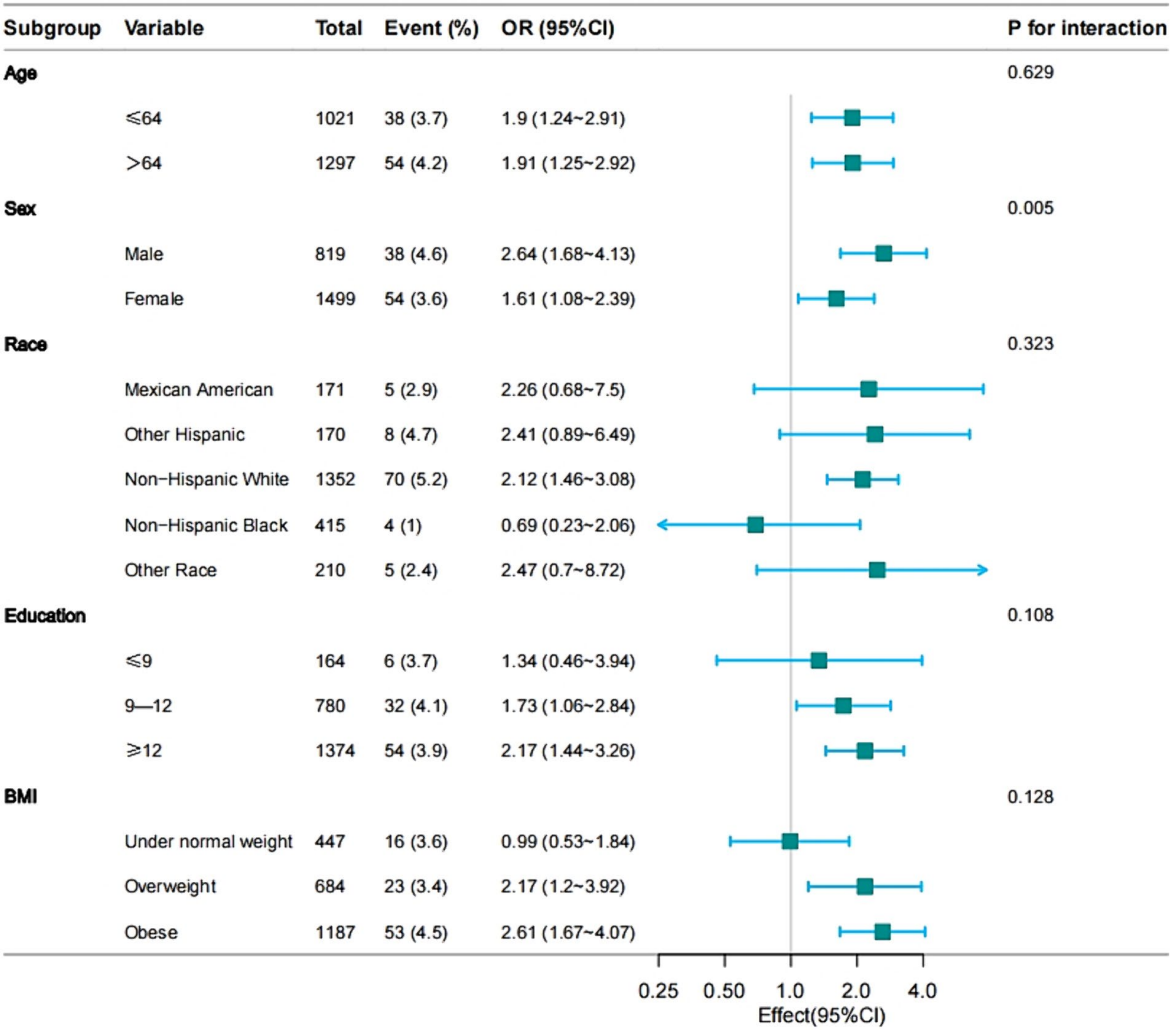
Effect size of osteoarthritis on the presence of PD in age, sex, race, education, BMI subgroup.

The subgroup analysis of the data is depicted in Figure 1. Stratified analyses were conducted to investigate if the association between various types of arthritis and Parkinson’s disease differed based on age, sex, race, education, and BMI. Furthermore, the results were validated for all subgroups except for sex, showing consistent findings across all subgroups without any interactions (Figure 2). The findings indicated a significant correlation between osteoarthritis and the risk of developing Parkinson’s disease, which was statistically significant (*p* < 0.001).

## Discussion

Arthritis, a common disease among the elderly and a trigger for systemic inflammation, has an unclear role in PD. Therefore, it is crucial to investigate the potential correlation between PD and OA from both clinical and social perspectives. Our study examining the relationship between OA and PD revealed a positive association between PD and OA, as well as other forms of arthritis, but not with RA. Through multivariate logistic regression analysis adjusting for confounding factors, we observed a positive correlation between PD and OA risk, while no association was found between RA and PD. Subgroup analysis further supported the stability of these findings.

In this cross-sectional study, we found a clear association between OA and a significant increase in the occurrence of PD. Individuals with OA had a 95 percent higher risk of PD compared to those without arthritis, while no association was observed between RA and PD. This contrasts with findings from previous studies (Liu et al., 2023; Zeng et al., 2024). The discrepancy between our study and others may be attributed to differences in sample populations. Furthermore, our focus on OA during data cleaning may have influenced the results on RA and PD, making the association with RA inconclusive. Our study also revealed a significant correlation between other types of arthritis, such as psoriatic arthritis, and PD. However, due to the limited sample size, further investigation on these types of arthritis was not feasible. Moving forward, larger sample sizes are necessary to explore the relationship between other forms of arthritis and PD.

Arthritis is a degenerative joint disease encompassing OA, RA and other forms. OA is the most prevalent joint disease, characterized by an increase in both systemic and local inflammatory cytokines that contribute to cartilage degradation and impact OA progression (Motta et al., 2023). OA is characterized by cartilage degeneration, synovitis, and changes in subchondral bone, triggering a peripheral inflammatory response. RA, a chronic autoimmune disease, is marked by persistent inflammation in synovial joints. The damage to articular cartilage involves various immune cells like NK cells, B lymphocytes, T lymphocytes, among others (de Lange-Brokaar et al., 2012; Li et al., 2022). Arthritis, a non-specific inflammatory condition, not only inflicts irreversible harm on the synovium but also may contribute to other immune-related disorders like inflammatory bowel disease, systemic lupus erythematosus, diabetes, Graves’ disease, and more (Tagoe et al., 2012; Houser and Tansey, 2017; Veronese et al., 2019). The dysregulation of immune homeostasis in patients with arthritis results in an increase in peripheral pro-inflammatory factors and a decrease in anti-inflammatory factors. This imbalance also impacts inflammatory factors and neurotransmitters, which can further enhance the interaction between immune cells and nerves through mutual regulation (Gao et al., 2022). Research indicates that inappropriate peripheral inflammation may trigger a neuroinflammatory response, leading to the disruption of the blood–brain barrier by inflammatory stimuli, potentially contributing to the development of central nervous system disorders (Huang et al., 2021). Moreover, cytokines released by peripheral inflammatory mediators involved in chronic inflammation can activate glial cells, exacerbating neuroinflammation and directly influencing the function of the blood–brain barrier as well as neurodegenerative processes (Sonar and Lal, 2018). Neuroinflammation is a crucial mechanism in the pathogenesis of PD, serving as both a key factor in disease onset and progression (Zhang et al., 2023). Inflammatory processes can lead to the degeneration of dopamine neurons, which significantly impacts individuals with PD (Lücking et al., 2000; Perry, 2004). Factors such as *α*-syn misfolding, immune gene polymorphisms, and mitochondrial dysfunction contribute to the development of neuroinflammation in PD patients (Isik et al., 2023). Autopsy studies have confirmed the involvement of microglia-mediated neuroinflammation in the pathogenesis of PD (McGeer et al., 1988). Natural killer cells present in PD patients have been found to effectively degrade *α*-syn aggregates. Depletion of natural killer cells in *α*-syn mouse models has been shown to worsen *α*-syn aggregation and promote dopamine neuron degeneration, suggesting a potential link between peripheral inflammation and PD development (Earls et al., 2020). In addition, elevated levels of systemic inflammatory cytokines have been identified as playing a significant role in the development of PD (Tansey et al., 2022). C-reactive protein (CRP), a key inflammatory marker for arthritis, has been found to have a positive correlation with the prevalence of PD in studies (Qu et al., 2023).

Research indicates that the increase of TNF-*α* and IL-6 in the bloodstream is also positively associated with PD, suggesting that the elevation of peripheral pro-inflammatory factors may be crucial in the pathogenesis of PD (Chen et al., 2008).

While the root cause of the differing associations between osteoarthritis and Parkinson’s disease remains unclear, this study possesses several strengths. Firstly, a significant advantage lies in the utilization of a large and representative sample of Americans gathered through continuous NHANES cycles, enabling the examination of the relationship between osteoarthritis, other forms of arthritis, and PD. Additionally, the potential neuroprotective role of smoking in PD, as indicated by previous studies (Gale and Martyn, 2003; Ascherio and Schwarzschild, 2016), was considered through covariate adjustments, with smoking identified as a crucial variable. Nonetheless, it is important to acknowledge the limitations of our study. Data constraints may have led to missing information, potentially stemming from a lack of awareness or delayed medical attention-seeking. Furthermore, the diagnosis of PD in individuals was primarily based on medication usage, raising concerns about potential biases from patients with other chronic illnesses or mental disorders also taking anti-Parkinson’s drugs. As a cross-sectional study, causal relationships cannot be definitively established, and despite adjustments for confounding variables, residual confounding remains a possibility. Thus, a comprehensive controlled trial is warranted. Moreover, the reliance on NHANES data restricted the ability to explore correlations between various types of arthritis and PD individually due to database limitations, necessitating further investigation to validate these findings.

## Conclusion

PD patients were positively correlated with OA. Among people with OA, there was a 95% increased risk of PD compared to people without arthritis. Therefore, when treating OA, attention should be paid to the increased risk of PD. In the meantime, further studies are needed to explore the link between OA and PD patients.

## Data Availability

Publicly available datasets were analyzed in this study. This data can be found at: https://www.cdc.gov/nchs/nhanes/index.htm.
